# Annual Museum

**Published:** 1895-08-03

**Authors:** 


### The Institutional Workshop.

### BRITISH MEDICAL ASSOCIATION.

### ANNUAL MUSEUM.

This exhibition was held in the Examination Hall on the Victoria Embankment, the rooms on the first floor being devoted to museum purposes. There were not perhaps quite so many stalls as at the Bristol meeting last year, but the show taken as a whole was a good one, and there were many very interesting exhibits. It must be said, however, that there was decided room for improvement in the general arrangement of the stalls, which were numbered only in the catalogue, and to trace any particular one was a matter productive of loss both of time and temper, which a very little method would have avoided.

The large central room (C) made a very imposing show, and some of the stalls were attractively got up. For descriptive purposes we propose to deal with the rooms alphabetically, rather than in the somewhat eccentric manner of the catalogue.

### Room A.

The first stall in Room A was devoted to the well-known Apollinaris Company's Natural Waters. Apollinaris itself, of which it has been said that "whoever drinks Apollinaris gains daily one repast the more and one indigestion the less," needs no comment to increase its popularity. The Friedrichshall Water, which is taken from the recently opened new spring, has been proved by analysis to be practically identical with the old, which was in use for more than half a century. Experiments carefully carried out have proved that no unpleasant effects are found to follow even a continuous taking of Friedrichshall Water.

Jeyes' Sanitary Compounds Company exhibit some new solvents for uric acid. Lysidine is claimed to have 60 times the solvent action of piperazine. Their coal-tar disinfectant, Jeyes' Fluid, is now prepared in a clear form; the 5 per cent. solution is almost absolutely transparent in water, and can be used for surgical instruments. The indispensable Automatic Toilet Box is a great novelty and a most ingenious invention. It is for holding sanitary papers. Only one leaf can be taken out at a time, and the box cannot be opened until all the papers have been used, thus a great economy may be effected and waste prevented. Migrainine and Nosophen are other new preparations worthy of notice.

A point to be specially noticed about the preparations of the Liquor Carnis Company is that they are made entirely from English and Scotch beef. "Virol," a food which is intended to supersede cod-liver oil, consists of beef marrow fat, taken from marrow bones sawn in sections. The marrow is dissolved at a very low temperature and combined with raw eggs, of which the shells are also used when dissolved by lemon juice. A special form of this "Virol sans Sucre" is prepared as a diabetic food. Another speciality to be noted on this stall was a preparation intended as a palatable substitute for cod liver oil. "Marrol" is bone marrow, plus a carbohydrate—extract of malt—in physiological proportions. It is not so sweet as "Virol," and is said to have considerable nutritious value.

Standard Malt Extract Company (Limited).—Diastol is a perfectly new preparation containing a large percentage of diastase with no carbohydrates. It is a very thin fluid, and can be readily taken by patients who resent the usual thick preparation of Maltine. Malt extract with cod-liver oil is a useful preparation and well tolerated. It has an agreeable odour. The cardboard boxes in which these preparations are packed are a feature in the exhibit. The liquid malt extract is thinner than the ordinary malt extract, though it contains a high percentage of diastase.

Curry and Paxton showed many varieties of ophthalmoscopes, and spectacles and eye-glasses of the newest and most approved patterns.

J. Defries and Sons have a fine exhibit of Pasteur (Chamberland) filters for table use as well as some other varieties for fitting to service pipes or cisterns. Their Equifex spray apparatus, for disinfecting objects without the use of steam, is a useful innovation. Dr. Scurfield's portable air-testing apparatus is an effective means for roughly estimating the ventilation of a room.

Mackey, Mackey, and Co. show their beautiful bismuth preparations. Their gelata are also of excellent quality, especially that containing eucalyptus oil (Gelatum eucalypti). This preparation is used for smearing the hands before a surgical or obstetrical operation, thus ensuring antiseptic conditions. When the hands are washed afterwards the jelly works into a fine lather, and plays the double part of an antiseptic and soap.

Sulphur and Chalybeate Waters were shown by the Harrogate Corporation, who also had on view a good collection of photographs of their town, and attractively got up handbooks.

Callard and Co. exhibited a great variety of diabetic foods, many of which, notably their almond meal and meat biscuits and Parmesan cheese straws, with many other sponge cakes and shortbreads, had a most appetising appearance and flavour.

Burgoyne, Burbridges, and Co. exhibit some fine samples of antiseptic dressing, Red Cross antiseptic wool and lint. Burgoyne chlorobrom is an excellent preparation, and likely to become exceedingly popular.

Macfarlane and Co. show samples of their celebrated specially purified chloroform and ether. Both of these anæsthetics are now well known to the profession, and are largely used in several London hospitals. Their antiseptic dressings, ligatures, &c., are all of first-rate quality.

### Room B.

Ferris and Co. publish some very excellent medical visiting lists for combined use with a ledger, without the employment of a daybook, and thereby reducing bookkeeping to its simplest terms. The Ever-ready Caddy of surgical dressings is a most valuable possession for the general practitioner or casualty surgeon, containing as it does, in the most handy form, every variety of dressing, bandage, or strapping that ingenuity can suggest. Dr. Mitchell Clark's sputum case is a compact box containing every possible requisite for testing the sputum of phthisical patients.

The Norwegian Milk Condensing Company showed the "Viking" brand of unsweetened condensed milk. The process followed was discovered by Dr. Olav Olsen, of Christiania. The milk is guaranteed absolutely free from bacteria, and it will keep for any length of time in any climate so long as the tins are unopened. It is therefore particularly valuable for use abroad. Another exhibit meriting notice was "Gaskin's pure soluble cocoa," for which the above firm are agents. This is an excellent cocoa, nutritious and easily digestible, and its strength may be guessed by the quantity (half a teaspoonful) which is sufficient for a breakfast cup.

Messrs. Rebman were in possession of a stall plentifully supplied with medical literature. We noticed among other volumes "Gynæcology (Medical and Surgical)," edited by J. M. Baldy, M.D.; and a new work, edited by Dr. Norris,

"Obstetrics." Also "Diseases of Children," by Louis Starr, M.D.

Messrs. Cassell and Co. were well to the front with their medical and clinical manuals, and we also noticed Mr. Treves' "System of Surgery," and the new numbers of the enlarged "Practitioner."

J. H. Montague has some excellent samples of surgical instruments of the latest design and pattern, nearly all of metal throughout, and intended for aseptic surgery. Carter Braines' Improved Junker's chloroform apparatus has many improvements on its prototype, and no liquid chloroform can escape down the exit tube. A new pattern of ear syringe deserves attention, the tympanic membrane cannot be damaged, nor can the outflowing lotion trickle down the face or neck. A new form of dressing syringe, with two finger rings, as used in Guy's Hospital, Dr. Carre's transfusion box, and a variety of light aluminium splints form part of a most interesting exhibit.

Armour and Co. show some fine samples of animal ferments, in the dry as well as in the fluid form.

Cooper and Co. show for the first time an innovation in the manufacture of cachets for large doses. They are made narrower than usual, but correspondingly thicker, the act of swallowing being thus greatly expedited, and, further, the name of the drug is printed instead of being embossed on the surface of the cachet. Their liquor quinine salicylatis is a good preparation of quinine.

Samples of aerated waters were to be found on the stall of Messrs. Idris and Co., together with exhibits from chemical and bacteriological laboratories, including cultures obtained from water before and after aeration, and exhibitions of various methods of analysis, &c. On this stall also were exhibits from the Viking Food and Essence Company of meat essences and invalid delicacies.

### Room C.

Borroughs, Wellcome, and Co.—This celebrated firm, as usual, occupy the most prominent position in the museum. Among the most important of their preparations may be mentioned emol-keleet, which is shown in all stages of manufacture. It is probably destined to replace Fuller's earth, as a dusting powder, and as an application for inflamed and weeping surfaces. It is an impalpable mineral powder, and constitutes an excellent vehicle for antiseptic agents. The tincture tabloids (before noticed in this journal), tea tabloids, anti-diphtheritic serum in the dry form, various compressed drugs, soloids carrying antiseptic agents highly soluble in water, all form interesting features in this magnificent exhibit. In connection with their famous medicine cases and chests this firm also exhibit that which once belonged to the late Dr. Livingstone, also the famous Stanley "Congo" chest, and several others of historic interest. The Jenner exhibit includes besides many of the original drawings of the vaccine pustules, and the very armchair in which the great discerner died.

Allen and Hanburys (Limited) had an interesting exhibit, prominent in which might be noticed their famous castor oil, and other preparations such as the "Perfected" cod-liver oil, soluble gelatine-coated pills, and preparations of animal extracts, notably "Thyroidin." This firm are also agents for the anti-toxins prepared by the British Institute of Preventive Medicine.

Arnold and Sons.—A novelty introduced by this firm is Dowding's Tongue Depressor, which is provided with a talc guard to prevent sputum or membrane being ejected into the face of the observer by diphtheritic patients. It is yet transparent enough to allow observation of the fauces. The Compactum Midwifery Bag for aseptic cases, containing pocket instruments and provided with leather purses as well as aseptic cases of Southey's tubes, are most fascinating exhibits. The new mechanical arm for amputations above or below the elbow seems almost endowed with life. With one of them the patient can carve and lift food to his mouth, as well as perform manual operations that require the use of jointed fingers, wrist, and elbow.

The Aylesbury Dairy Company showed specimens of their now famous "Humanised Milk," prepared in vacuum-stoppered bottles, of which some thousands are sold weekly, so valuable a substitute for mother's milk is this universally considered. Several other preparations we also noticed of sterilised milk and cream; and Muffler's Food, of special use for infants when able to digest nourishment of a more solid nature than milk.

Fairchild Brothers and Foster show samples of the first peptogonic powder imported into this country. The feature of this preparation is that if added to diluted milk in the correct proportion, a milk of the same percentage as the human variety is at once formed. This constitutes a cheap and valuable substitute for ordinary humanised milk.

Down Bros. show a magnificent collection of aseptic surgical instruments. A great feature of this exhibit is a series of bags for use in aseptic surgical gynæcology or midwifery. The instruments, dishes, &c., are all of metal, and can be at once sterilised by dry heat or steam. Such complete and portable bags have never before been exhibited. They mark an epoch in the evolution of aseptic gynæcology, and place Down Brothers in the enviable position of being without a rival in the manufacture of midwifery bags and requisites. Murphey's anastomosis buttons, for use after resection of a portion of the intestine, are also on view, and are of interest as another advance in surgical carpentry. Anæsthetic apparatus, with modification by Tyrrell, Silk, and Porrit form a considerable part of their exhibit.

At the Cerebos Salt Company's stall were to be seen that "Nutritive Salt" itself, which has the advantage of containing a substantial proportion of phosphates, besides being very palatable for table use, and also the "Cerebos Baking Powder," which also contains the mixed bran phosphates, and has special aërating qualities.

The Condal Water Company had specimens of their Natural Aperient Water, and next to them were to be seen the products of the Peat Industries Syndicate, including surgical and veterinary dressings, hygienic blankets, socks, &c. The special value of these consists in their power of absorption and prevention of decomposition, and extreme softness. They are also quite inexpensive.

John Wright and Co., of Bristol, had an imposing array of medical publications, amongst which we specially noticed "Diseases of the Upper Respiratory Tract, the Nose, Pharynx, and Larynx," by Dr. Watson Williams; Snell's "Miner's Nystagmus," and "Eye Sight and School Life," by the same author; Dr. G. Armauer Hansen and Dr. Carl Looft's "Leprosy; in its Clinical and Pathological Aspects"; "Herbal Simples," by Dr. Fernie; and a third edition of Mr. Hurry Fenwick's "Urinary Surgery." Messrs. Wright also were showing a very neat "Doctor's Visiting List" for 1896, compiled by Robert Simpson, L.R.C.P.

The Johannis Mineral Waters, of which there was a good show, are too well known to need comment.

Oppenheimer, Son, and Co.—This firm show drugs of 700 different formulæ prepared in the form of palatinoids. The active principles of many animal glands, such as the thymus, and suprarenal are offered in this agreeable form. Concentrated liquors are a speciality introduced by this firm, and their exhibit contains a magnificent series. Their cream of malt, with or without cod-liver oil, appears of first-class quality. A specimen of Ergole, containing all the active principles of ergot, is of special interest since it is claimed for it that it does not create nausea, or produce inflammatfon when hypodermically injected. A new atomiser on view at this stall is a marvel of efficiency; it is said to be capable of atomising olive oil, and it certainly looks as if it could. This firm are also agents for the supply of the anti-toxic serums prepared at the Pasteur Institute in Paris.

The special preparation of meat juice shown by Messrs. BRAND AND CO. as their last and best effort in the line for which they are so well known, deserves a word of notice. This juice is extracted cold by pressure, and therefore contains the albumen uncoagulated. It is a very pleasant preparation, and is taken in the proportion of a teaspoonful to a wine-glass of cold water. Among many and various forms of jelly and bouillon we noticed a beef broth, prepared for hospital use, sold in 1 lb. and ½ lb. tins, and providing a good and cheap food for the poorer classes of the population at a moderate price.

THOMAS CHRISTY AND CO. are exhibiting some specimens of hæmoferrum pilloids, containing the iron as directly obtained from blood, and a number of preparations of new drugs from South America, including the juice of Solanum Grandiflorum, said to be a specific for asthma.

NESTLÉ'S Swiss Milk and Food for children and invalids maintains its position as one of the most popular of infant foods, and was well represented at the stall devoted to these preparations.

PARKE, DAVIS, and CO., have a fine collection of animal extracts, among them being the extract of the pituitary gland in the form of tabloids and elixir. This is quite a new departure in the new therapy, and is supposed to be of value in certain nervous complaints. The extracts of brain, supravenal capsules, and testes have each attracted considerable interest in the exhibition. Hæmorrhoidal tubes with nozzle and collapsible body containing various ointments form part of a most interesting exhibit.

Natural mineral waters might be certainly said to abound in the exhibition, from which one may judge that their popularity is ever increasing. Messrs. INGRAM and ROYLE (Limited) exhibited specimens of their Æsculap and Vichy waters. The first-named water comes from a spring in Hungary, which is the freehold property of the company, the bottling and management being, therefore, carried on directly under English supervision. The Carlsbad mineral water is also now prepared in the form of powder for prescription in cases of chronic gastric catarrh, gout, diabetes, &c.

The SANITAS COMPANY show a large collection of their different hygienic and sanitary preparations.

FRY'S cocoa and chocolates are almost too much "house hold words" for mention, but their pure concentrated cocoa, which is scientifically prepared and extremely soluble, was, perhaps, the most important of their exhibits.

The MALTINE MANUFACTURING COMPANY had various preparations of malted barley extracts, supplied in conjunction with cod-liver oil, iron, cascara sagrada, &c. Especially we should mention their liquid peptonoids, a solution of the beef peptonoids powder, "entirely digested, with the addition of the minimum amount of alcohol necessary to make a stable compound." This and another preparation with coca are both very pleasant to taste, and have been found a successful combination.

### Room D.

ARTHUR AND CO. exhibit several novelties in the way of gold and arsenic preparations. The liq. auri. brom. arsen. mercuratis is quite a new departure in practical therapeutics, and may possibly prove of considerable service in the treatment of epilepsy. The unguentum hammamelidis co. is now acknowledged to be a first-rate ointment for hæmorrhoids. Tongue cloths made of Japanese paper are invaluable for laryngitseopists, and handkerchiefs made of the same paper are a useful substitute for the linen variety in cases of phthisis with much expectoration, since they are prepared with a powerful germicide and can be readily destroyed. Sedative snuff for influenza and catarrh, and iodo-tannin wine, a substitute for cod-liver oil, are both worthy of notice.

THE LEAMINGTON TOWN IMPROVEMENT ASSOCIATION showed specimens of the natural mineral water peculiar to Leamington and its neighbourhood, and some prettily got-up guide books with fascinating descriptions of its attractions.

W. MARTINDALE show some magnificent preparations of alkaloidal salts. Indeed, their exhibit is quite one of the features of the museum. Scopolamine is a new mydriatic with five times the effect of atropin, and, further, it has the advantage of causing no dryness in the throat. It is prepared in the form of a hydrobromate, and is very soluble. Cocain nitrate is also an innovation in the pharmacy of cocain. It will mix with nitrate of silver without decomposition, a most valuable property. Quin. hydrocloride sulphate is a new salt of quinine, very soluble in water (1 in 4), whereas the ordinary acid hydrobromate is only soluble to the extent of 1 in 8. Guaiacol is a new synthetic production. It is consequently absolutely pure, and, further, it is in a beautiful crystalline form. Vinum emetine is made by dissolving emetine in detannated sherry. It has the therapeutic properties of the vinum and is more reliable. The small collapsible tubes of ointments, used only in small quantities, such as unguentum atropinæ, are economical and handy.

THE LIVERPOOL LINT COMPANY had an extensive show of bandages and dressings of all sorts and descriptions. Domette, flannel, and calico bandages, strong and light, protective lint, absorbent wool, and every other appliance for the dressing of wounds, &c., were on view at this stall. A "splint" padding, a combination of absorbent wool and carbolised tow, made up as a thick and very soft pad between layers of gauze, formed a particularly useful dressing. The goods manufactured by this firm are always to be recommended.

WARRICK BROTHERS show samples of their new famous iron jelloids, containing the protosalts of iron in a most assimilable form.

The "beef jelly" exhibited by the BOVRIL COMPANY is a speciality to be noted. It is packed in glass, thus avoiding any contact with metal, and is an excellent invalid food. Of Bovril itself, as generally known, it is hardly needful to speak. The many compressed forms in which "emergency foods" have been supplied to the various Arctic exhibitions were shown, and the ingenuity of their preparation is really matter for wonder. Those who have visited the Bovril factory will not have failed to be impressed by the excellent management which is there apparent, and of Bovril preparations it can be said that their popularity can only increase with a further knowledge of the methods employed in their production.

JOHN WEISS AND SON shew a magnificent collection of surgical instruments, chiefly made of metal throughout, for antiseptic surgery. Some of the smaller instruments for eye and aural surgery have aluminium handles for the sake of lightness. They are of most beautiful finish and are fitted in light metal cases. One of their hypodermic syringes is so small that case and all it is little bigger than an ordinary double sovereign purse, although it contains four small tubes for hypodermic injections. The exhibit also comprises some primary and secondary batteries for laryngoscopical purposes. The secondary batteries may further be employed for driving a motor with which to drill bone, teeth, &c., or combined with a galvanic cautery for removing polypi, &c. The various instruments used by Mr. Victor Horsley in his brain and spinal operations are made by this firm and form an interesting part of the exhibit.

VAN ABBOTT AND SONS show a variety of diabetic foods in a more palatable form than is often the case with such preparations. Their chocolate, cocoa-nut, and almond biscuits are practically as nice as the ordinary varieties containing a large amount of sugar. Their bran biscuits should answer well in cases of chronic constipation.

The Scientific Press (Limited).—The chief feature in the stall devoted to the publications of this firm is Mr. Henry C. Burdett's "Hospitals and Asylums of the World." Its four large volumes, in which are to be found all the information worth having on hospital matters, and portfolio of plans, attract attention at once. Amongst other volumes we noticed Dr. Withington's interesting "Medical History from the Earliest Times," Dr. Percy Lewis's "Theory and Practice of Nursing, "Mental Nursing" by Dr. William Harding, &c. The reports of the Johns Hopkins Hospital Medical School were of special interest to medical men, and also the publications of the W. T. Keener Company, of Chicago, for both of which series the Scientific Press are the sole agents in the United Kingdom. The well-known account books designed for the use of hospital secretaries and managers by Mr. H. C. Burdett, and the same author's "Hospital and Charities Annual," now in its sixth year of publication, and universally admitted to be the standard work on the subjects it deals with, must also be noticed. "Science Progress," which owes its being to this firm's enterprise, and of which the *Pall Mall Gazette* recently said, "It has now well entered into its stride as the representative of advanced science," must not be omitted from the list.

The Sanitary Wood Wool Company exhibit many antiseptic dressings, sponges, gauzes, &c.

Messrs. Evans, Lescher, and Webb show as a speciality their preparations of sandal-wood oils.

Among the exhibits of Messrs. Wyley's stall we specially noticed various forms of compressed pellets, pearl-coated pills, flexible and soluble gelatine capsules, &c. An exceedingly neat pellet case commanded admiration, and also an ingenious ointment tube for ophthalmic use.

Leslies (Limited) showed varieties of dressings and plasters, amongst the latter we particularly noticed a holland backed one, of extra strength and very pliable and soft.

### Room E.

Messrs. H. K. Lewis exhibited a goodly number of medical and nursing publications, amongst which should be mentioned "Notes on Medical Nursing," by the late Dr. James Anderson, edited by E. F. Lamport; "A Handbook of the Theory and Practice of Medicine," by Dr. F. T. Roberts; Dr. Soltau Fenwick's "Dyspepsia of Phthisis"; "The Extra Pharmacopæia," by William Martindale, F.C.S., and W. Wynn Westcott, M.B.; "Diseases of the Nose and Throat," by Dr. F. de Havilland Hall, M.D.; and Dr. Louis Parke's "Hygienic and Public Health."

Specimens of the "Catley Abbey" Natural Seltzer Water were shown by the firm of that name, obtained from springs at Digby, Lincolnshire, so far the only natural seltzer spring known to exist in the British Isles. A special value in cases of gout and rheumatism is claimed for this water. As a "British product" the Catley Abbey Natural Seltzer Water ought to obtain good patronage from the British public.

Price's Patent Candle Company had an exhibition of their many well-known specialities—glycerine, soap, &c.

Anglo-Swiss Condensed Milk Company showed their "Ideal Milk," enriched 20 per cent. by the addition of pure cream, sterilised and unsweetened, and other well-known milks of the Milkmaid Brand.

The Flitwick Chalybeate Company had sample of "Wikko," a sparkling non-alcoholic preparation from the natural Chalybeate Water of Flitwick, in Bedfordshire. The taste of the natural water is somewhat rough, but the sparkling beverage made therefrom forms a pleasant drink and a palatable medium for taking iron.

An ingenious "Container" for medicated bandages, exhibited by Fassett and Johnson, agents for Seabury and Johnson, New York, deserves a word to itself. It is made of wood fibre package, into which is incorporated an antiseptic; it is coated within with antiseptic lacquer, and by overlapping is rendered perfectly air-tight. Its freedom from breakage is also an advantage.

Malto-Peptone Malt Extract and Jelly, with other preparations, including cod-liver oil, are the most important of the exhibits of the Malto-Peptone Company. The jelly with cod-liver oil is specially useful for children and invalids, as hardly a trace of the flavour of the oil will be found if the necessary quantity is cut up and swallowed in small pieces.

The British Castor Oil Co. keep their excellent tasteless castor oil to the fore.

### King's College Section—Rooms Nos. 27, 28, 29, 34, and 36.

Here in the quadrangle we noticed one of those excellent portable hospitals of the Berthon Boat Company (Limited), of which full notice appeared in our issue of last week.

Entering King's College, and passing through the vestibule, which was tastefully decorated with flowers, and where we noticed the ever-useful bookstall of Messrs. W. H. Smith and Son, with a plentiful supply of medical and other journals, we found ourselves in the large hall, which is being used as the general reception room, and which might very well be called the general utility department. The centre of the room was quite clear, whilst around the open space were ranged counters, behind which an army of Commissionaires presided. Here were the post and telegraph offices, as well as a counter where letters and telegrams are kept for members. Large placards arranged over the counters denoted where tickets for the different entertainments and things worth seeing in London could be obtained. In fact, every convenience seemed to be provided to save trouble to the medical practitioner who is unacquainted with town.

Passing up the stairs we come to the exhibit (room 36) of the Ceres Automatic Letter and Card Files, interesting as showing how space can be saved by ingenuity, and how useful a writing desk or table can be made with the exercise of a little brain power.

In room 28 were to be seen Messrs. Down Bros.' excellent appliances for the operating theatre. The aseptic furniture for out-patients' rooms at hospitals and for hospital wards was everything that could be desired.

Room 29: Here, again, were some more excellent fittings and appliances for the operation theatre, shown by Messrs. Allen and Hanburys (Limited), amongst which we particularly noticed a consulting-room couch designed by Dr. W. H. Marriner, also one designed by Dr. J. R. Whait, which has the advantage of being adapted for gynæcological and surgical work.

Room 34: Gustav Hermanni here shows samples of Ichthyol, a peculiar close combination between sulphur and carbon, the feature of which is that it introduces both elements into the system in a pure and, at the same time, soluble form. Professor Dr. Baumann, of Berlin, reports very favourably upon this drug.

Space does not permit us to more than mention the exhibits of T. J. Syer and Co., operating table, &c.; Mr. L. W. Bickle's improved bed; Brigade-Surgeon Temple Wright's bungalow and ambulance carts; and Mr. John Jones's sanitary appliances.

